# Quantifying Carotid Stenosis: History, Current Applications, Limitations, and Potential: How Imaging Is Changing the Scenario

**DOI:** 10.3390/life14010073

**Published:** 2024-01-01

**Authors:** Luca Saba, Roberta Scicolone, Elias Johansson, Valentina Nardi, Giuseppe Lanzino, Stavros K. Kakkos, Gianluca Pontone, Andrea D. Annoni, Kosmas I. Paraskevas, Allan J. Fox

**Affiliations:** 1 Department of Radiology, University of Cagliari, 09042 Cagliari, Italy; dr.scicoloneroberta@gmail.com; 2 Neuroscience and Physiology, Sahlgrenska Academy, 41390 Gothenburg, Sweden; elias.johansson@neuro.gu.se; 3 Department of Cardiovascular Medicine, Mayo Clinic, Rochester, MN 55905, USA; nardi.valentina@mayo.edu; 4 Department of Neurologic Surgery, Mayo Clinic, Rochester, MN 55905, USA; lanzino.giuseppe@mayo.edu; 5 Department of Vascular Surgery, University of Patras, 26504 Patras, Greece; kakkos@upatras.gr; 6 Centro Cardiologico Monzino IRCCS, Via C. Parea 4, 20138 Milan, Italy; gianluca.pontone@cardiologicomonzino.it (G.P.); andrea.annoni@ccfm.it (A.D.A.); 7 Department of Biomedical, Surgical and Dental Sciences, University of Milan, 20122 Milan, Italy; 8 Department of Vascular Surgery, Central Clinic of Athens, 14122 Athens, Greece; paraskevask@hotmail.com; 9 Department of Medical Imaging, Neuroradiology Section, Sunnybrook Health Sciences Centre, University of Toronto, Toronto, ON M4N 3M5, Canada; ajfox@uwo.ca

**Keywords:** carotid disease, carotid stenosis, carotid endarterectomy, near-occlusion, vulnerable carotid plaque, plaque-RADS, artificial intelligence, DSA, CTA, MRA

## Abstract

Carotid artery stenosis is a major cause of morbidity and mortality. The journey to understanding carotid disease has developed over time and radiology has a pivotal role in diagnosis, risk stratification and therapeutic management. This paper reviews the history of diagnostic imaging in carotid disease, its evolution towards its current applications in the clinical and research fields, and the potential of new technologies to aid clinicians in identifying the disease and tailoring medical and surgical treatment.

## 1. Introduction

Long before the intricacies of vascular medicine were understood, ancient physicians like Hippocrates, who referred to the carotids as “soporales”, recognized their significance for cerebrovascular events [1,2]. It took centuries of medical evolution, with contributions from pioneers like Virchow and others, to elucidate that carotid atherosclerosis was a key player in cerebrovascular incidents [3].

The journey of understanding carotid disease underwent a significant leap in the 1950s with Miller Fisher’s reports of carotid plaque with associated thrombus and intraplaque hemorrhage and the observation that most symptomatic patients had at least 75% stenosis, or a luminal diameter of 1 mm or less [4,5]. His findings served as a foundation for the multiple early reconstructive surgeries/endarterectomies for stroke prevention performed in the 1950s [2,3,6]. However, a crucial question arose: who should be treated? It was evident that not every patient with carotid atherosclerosis required intervention. Biomarkers were needed to stratify patients and determine who would benefit from revascularization procedures [6].

By the early 1980s, vascular studies of the carotid arteries primarily relied on instrumental invasive angiography, done at first with direct puncture of the carotid or brachial arteries, then femoral catheterization, with thromboembolic stroke risk demanding a high level of precaution, because of arch manipulation to achieve selective catheterization of the common carotid artery. Imaging was recorded with X-rays, at first through film-screen serial film changers and then with digital subtraction angiography (DSA) systems [7]. The technologies of computed tomography (CT), magnetic resonance imaging (MRI), and ultrasonography (US) were not yet sufficiently advanced to effectively study the carotid artery as we can today. Therefore, the search for a biomarker had to be conducted with the only tool available at the time: angiography. As a purely luminographic method, angiography could only reveal narrowing or morphological changes, such as ulcerations [8].

It was in this period that the major trials of the North American Symptomatic Carotid Endarterectomy Trial (NASCET) [9,10] and the European Carotid Surgery Trial (ECST) [11,12] began and were stopped far ahead of the planned time because of the compelling surgical benefit for the most severe stenosis cases. There had been previous smaller studies of carotid stenosis, including the randomized joint study of 1970 [13], that failed to show positive results for surgery.

Both NASCET and ECST used the degree of stenosis as an anatomical biomarker for therapeutic indications. Their findings provided a pivotal foundation for decision-making in the management of carotid artery disease, setting the stage for the evolution of imaging and its impact on the field [9,10,11,12].

## 2. Models for Assessing the Degree of Carotid Stenosis

### 2.1. The NASCET

The North American Symptomatic Carotid Endarterectomy Trial (NASCET) [9,10,14], conducted between 1987 and 1996, randomizing 2885 patients, with 1415 cases in the surgical arm, stands as a cornerstone for the understanding and management of carotid artery disease. This landmark study was designed to evaluate the use and effectiveness of carotid endarterectomy (CEA) in patients with symptomatic carotid stenosis, including a focus on the degree of stenosis as an indication for intervention.

**Figure 1:**
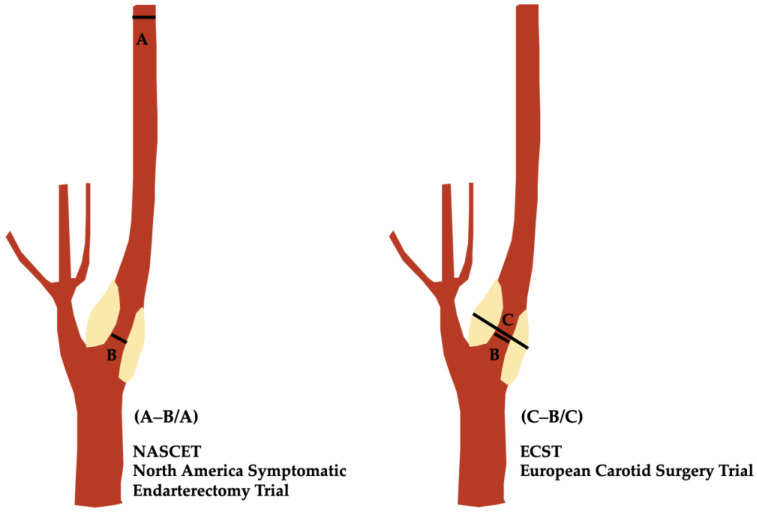
Schematic representation of ICA stenosis, illustrating NASCET and ECST measurement methods. B, luminal diameter at the site of maximal narrowing. A, diameter of the normal distal ICA well beyond the bulb, where the artery walls are parallel. C, diameter of the estimated original width of the ICA at the site of maximal narrowing.

NASCET enrolled patients with symptoms of transient ischemic attack (TIA) or minor stroke and carotid stenosis ranging from 30% to 99%. The trial’s methodology was rigorous, employing DSA to assess the degree of stenosis. One of the study’s significant contributions was the use of a consistent method to calculate the degree of stenosis, which became widely adopted after the publication of the NASCET results, conditional upon two steps:

(1) To seek near-occlusion if present and, when found, not to apply the method;

(2) To identify two critical points on an angiographic image (Figure 1): (a) the narrowest diameter of the internal carotid artery (ICA) stenosis; (b) the normal diameter of the ICA well beyond the ICA bulb, where the arterial walls are parallel and unaffected by atherosclerotic plaques.

The degree of stenosis is then calculated using the following formula:

**Degree of Stenosis (%) = [1 − (Minimal luminal diameter/Diameter of normal distal ICA)] × 100**

This formula is a simple arithmetic formula whose accuracy depends upon the correct measurements and respecting the recognized pitfalls. Of note, the real stenosis measurement is the numerator of the ratio, i.e., the minimal luminal diameter, whereas the measurement risks are based mostly on the denominator.

According to the NASCET criteria, stenosis is categorized as mild (<30%), moderate (30–69%), or severe (70–99%). Complete occlusion is indicated when the stenosis is 100% [9]. Later, moderate groups were subdivided into low-moderate (30–49%) and high-moderate (50–69%) [10].

The 1991 results of NASCET [9] were groundbreaking, showing the high rate of stroke for patients with high-grade stenosis (70–99%) and the reduced risk of ipsilateral stroke after CEA. Specifically, the study found that in this group, the procedure reduced the absolute risk of stroke by 17% over two years. In contrast, for patients with high-moderate stenosis (50–69%), the benefit was more modest, and for those with low-moderate stenosis (30–49%) the surgery did not offer any significant advantage over medical therapy alone [15].

NASCET had profound implications for clinical practice. The study provided clear guidelines for patient selection for carotid endarterectomy, advocating for surgery in patients with high-grade symptomatic stenosis. It also underscored the importance of accurate measurement and classification of stenosis severity.

Moreover, NASCET set a precedent for the standardization of imaging techniques and the quantification of carotid stenosis. Its method of calculating stenosis became a benchmark for clinical and research settings, facilitating consistency in diagnosis and treatment decisions.

### 2.2. The ECST

Parallel to the NASCET trial, the European Carotid Surgery Trial (ECST) [11,12,14] from 1981 to 1994 randomized 3024 patients, 1811 enrolled in the surgical arm. It, too, aimed to evaluate the efficacy of carotid endarterectomy in patients with symptomatic carotid stenosis, similarly to NASCET.

ECST enrolled patients who had recently experienced symptoms such as TIA, minor stroke, or retinal ischemia. The trial utilized angiography for the assessment of stenosis but calculated by comparing the narrowest diameter of the stenotic segment to the measured, imagined, estimated and unseen original diameter of the carotid bulb covered by plaque (Figure 1), according to the following formula:

**Degree of Stenosis (%) = [1 − (Minimal luminal diameter/estimated carotid bulb)] × 100**

This estimation was measured from the presumed diameter of the affected ICA bulb, which represents an aberrant condition given its size being larger than its source (the common carotid artery), as well as the location for most stenoses of the cervical ICA.

For ECST, 70–99% defines severe stenosis, 30–69% moderate and 0–29% mild.

Given the formulas, it is clear that any stenosis calculated by the ECST method will display a higher percentage since it is matched against the bulb as opposed to the unaffected ICA beyond the bulb according to the NASCET methodology.

Discrepancies between the results of these two workgroups for NASCET and ECST can be explained through their different methodologies: an important contribution came from Rothwell [16], who worked to reconcile basic percentage stenosis differences between NASCET and ECST (Table 1). For example, 70% stenosis by ECST criteria corresponded to approximately 50% stenosis by NASCET criteria, while 70% stenosis by NASCET was approximately 80% by ECST.

Therefore, while ECST did show that carotid endarterectomy reduced the risk of stroke in patients with severe stenosis (70–99% ECST), this group contained subjects with less severe stenosis than in the NASCET study and, consequently, the absolute risk reduction of stroke by CEA in ECST was less than in NASCET.

The contributions made by ECST extended beyond its findings on the efficacy of endarterectomy. It emphasized the importance of methodological consistency in measuring carotid stenosis and, through the discrepancies in results compared to the NASCET trial, raised awareness of the potential variability in results based on percentage ratio when the denominator is not standardized. The trial also underscored the need for a comprehensive approach in managing carotid artery disease, considering not only the degree of stenosis but also the patient’s overall clinical profile and risk factors.

### 2.3. The Direct mm Measurement

Improvements in technology and the development of iso-center computed tomographic angiography (CTA) have led to high quality three-dimensional (3D) reformatting, high-level vascular imaging, computer workstation infinite directions, and inherent calibrations with accurate metrics. All this is done in a very short time with no stroke risk as in prior standard catheter angiography, and without multiple X-ray exposures for each of the multiple directions for each studied vessel (now a thin beam is applied once to the scanned region). Multiple views are made from the one computer set [17]. The pitfalls of % ratio stenosis, that were mainly from the denominators for ratio calculations, for NASCET [9,10,15] and ECST [11,12], are no longer needed. This advance from Bartlett et al. [18] allowed a return to the disease description principles of Miller Fisher in the 1950s [4,5] that pointed to stenosis diameter as a crucial marker for management.

Bartlett’s team conducted a comprehensive analysis, involving 268 carotid arteries examined by two neuroradiologists. They measured the narrowest portion of each carotid stenosis in millimeters from axial source images, with multiple other reformatted views available for interpretation. For each internal carotid artery, except in suspected near-occlusions, NASCET-style ratios were also calculated. Their findings indicated excellent interobserver agreement, with correlation coefficients ranging from 0.78 to 0.89.

A crucial aspect of Bartlett’s method is its ability to confidently assess stenosis from source images, even in the presence of calcification. The study showed a linear relationship between mean percent stenosis and mean millimeter stenosis, correlating 1.3 mm to a 70% and 2.2 mm to a 50% NASCET-style ratio. This method of defining severe stenosis (70% or greater NASCET) exhibited high sensitivity, specificity, and negative predicted value.

Not only was the direct mm approach an advance from the ratio methods, eliminating the errors of faulty denominators (i.e., the reference vessels) and hence reducing reliance on ambiguous and often inaccurate mathematical estimations of carotid anatomy [18,19], it was also revealed as a good predictor of the cross-sectional area obtained from pixel counting [20].

As imaging techniques continue to evolve, approaches like Bartlett’s, published in 2006, have offered glimpses into a future where assessments are increasingly accurate, reproducible, and tailored to individual anatomical variations.

The stenosis measured by the different diameter-based methods is the same, but each technique “translates” the stenosis into its own “standards” based on their mathematical calculations. Since there is basically a linear relationship between these methods, data can be converted between methods [16,19,21,22].

**Figure 2:**
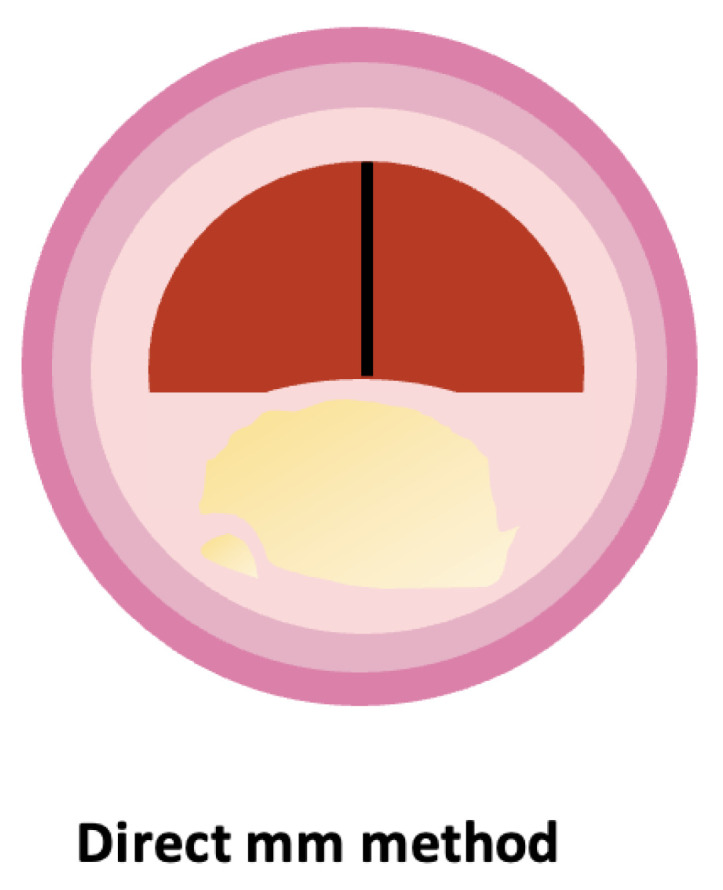
Schematic representation of the direct mm method.

Figure 2 is a schematic representation of the direct mm method.

## 3. Applications

### 3.1. The Guidelines and Use of NASCET: Endorsement from the Societies

As highlighted above, the results from both the NASCET [9,10] and ECST [11,12] trials showed the effectiveness of revascularization in reducing the risk of strokes and mortality in patients with a severe degree of carotid artery stenosis. These studies primarily utilized the percentage of stenosis in the ICA as the principal metric, offering a clear and reproducible method to determine a patient’s risk of stroke and distinguish between surgical and non-invasive treatment options.

As time has passed, to address the constraints of DSA-based measurements, new metrics have emerged, for instance area-based narrowing [23] or supplying the exact smallest diameter values [18], presenting an alternative view of obstructions created by plaques, factoring in all the 3D anatomical data available.

Given the lack of any guideline-backed threshold values for these newer techniques, the NASCET approach is still the prevalent method and the most frequently referenced in the guidelines, as Abbott and colleagues have found in their comprehensive review [24].

Recently, in 2023, the ESCR guidelines still recommended using the NASCET ratio for quantifying the degree of stenosis, to standardize the denominator, but also documenting the narrowest mm stenosis diameter for clinical and research purposes [25].

In any case, it remains crucial to always declare the method being referenced when indicating the degree of stenosis, which, as we have seen, could be a potentially significant confounding factor if not done [26].

### 3.2. Ultrasound Application

The study of carotid artery stenosis using US, particularly to grade and assess, has been a subject of extensive research and clinical interest as an alternative evaluation without the stroke risk inherent to angiography.

Using US, assessment according to NASCET criteria poses challenges in practical settings, namely high bifurcation, severe tortuosity, extreme plaque length, and acute thrombus, which can be difficult to visualize with B-mode imaging since it is weakly echogenic [27,28].

In response to these challenges, ultrasound technology, particularly the measurement of intrastenotic peak systolic velocity (PSV), has been explored as a surrogate parameter for evaluating carotid stenosis. PSV, a Doppler ultrasound measurement, representing the highest blood flow velocity during systole, is obtained by placing a Doppler probe on the carotid artery and measuring the blood flow velocity at the level of highest stenosis. The rationale behind using PSV as a surrogate marker is based on the principle that, as the arterial lumen narrows due to stenosis, blood flow velocity increases. Therefore, higher PSV values are generally indicative of more significant stenosis [29,30].

Early in the 1980s, historical comparisons with angiographic series were done that produced comparisons to percentage stenosis, before the NASCET and ECST methods’ discrepancies and pitfalls were understood [31]. After the major trials, many attempts to standardize ultrasound criteria for describing the degree of stenosis have been made, with conversion tables between NASCET and ECST methods and PSV [30,32,33,34]. For instance, a PSV above 230 cm/s is often considered indicative of stenosis of 70% or more, as per NASCET standards (Table 2) [30].

However, it is crucial to acknowledge that this association is indirect. PSV measurements do not provide a morphological assessment in line with NASCET rules, which specifically require visualization of certain arterial segments [30,35,36,37,38].

The reliance on PSV as a surrogate marker for carotid stenosis introduces several limitations. Firstly, PSV measurements can be influenced by factors other than the degree of stenosis. Variations in cardiac output, blood pressure, and even contralateral carotid occlusion can affect PSV readings. Another limitation is the potential for interobserver variability in PSV measurements. Ultrasound is operator-dependent, and differences in probe placement, angle of insonation, and interpretation of the results can lead to variability in measurements. This inconsistency can be problematic, especially when making critical clinical decisions based on these values [39,40,41].

Furthermore, PSV as a standalone measurement does not provide a complete picture of the morphological characteristics of the stenotic lesion. Information about plaque composition, surface characteristics, and presence of ulceration, which are essential in assessing stroke risk and guiding management, are not discernible from PSV measurements alone [40].

The reliance on PSV also introduces a risk of oversimplification. Carotid stenosis is a complex pathological entity, and reducing its assessment to a single hemodynamic parameter may overlook the nuanced understanding required for optimal patient management [40].

Therefore, PSV measurements offer a non-invasive, accessible, and quick method for evaluating carotid stenosis, and have important value in the era of angiography with associated risk of perioperative stroke; today, with angiography replaced by highly accurate, quick, stroke-risk-free CTA, the statistical justifications for PSV without angiography seem out of date for stenosis quantification as a substitute for angiography [40].

## 4. Current Limitations

### 4.1. Projectional Artifacts and Anatomy

The interpretation of angiographic images in assessing carotid artery stenosis is subject to a variety of factors, including projectional artifacts, anatomical variations, the larger carotid bulb, and, most importantly, lack of attention to the recognized pitfalls of deriving % ratio stenosis, whatever the method [42,43,44].

**Figure 3:**
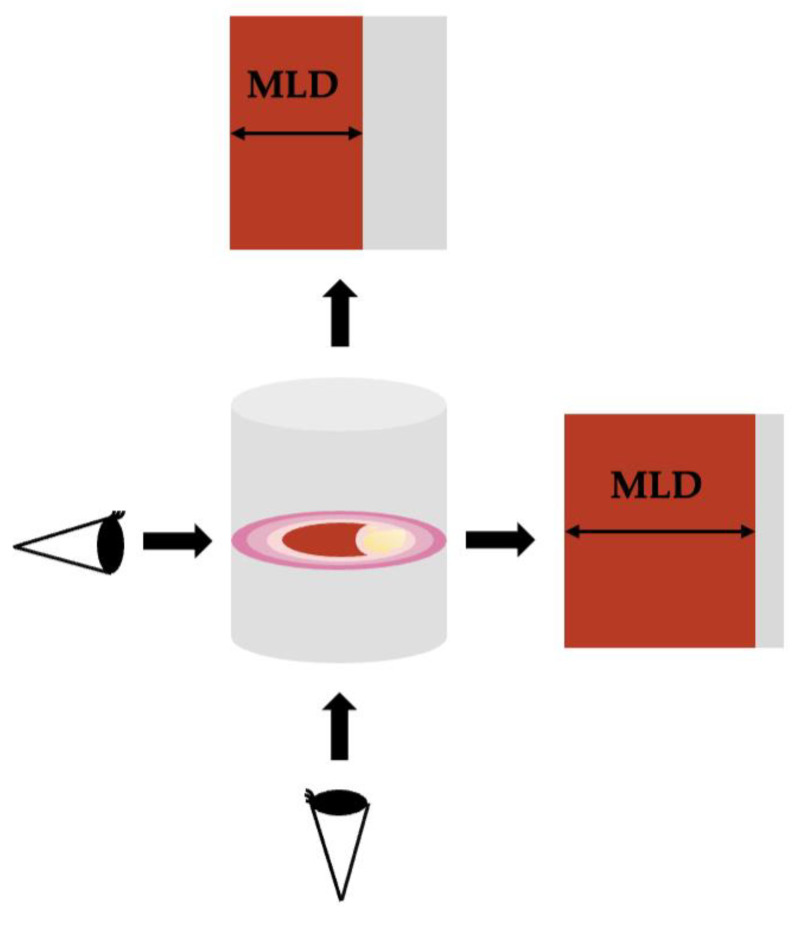
Drawing illustrating the projectional artifact. According to the viewpoint, the minimal luminal diameter (MLD) can appear smaller (larger stenosis) or bigger (smaller stenosis).

Projectional artifacts are a phenomenon inherent to angiography, where the two-dimensional representation of a three-dimensional structure can lead to misinterpretation. In the context of carotid stenosis, this becomes particularly salient when considering the morphology of plaques; specifically, a plaque that is eccentric or irregular in shape can appear markedly different in varying angiographic projections. For instance, in one view, the plaque may seem to protrude significantly into the lumen, suggesting a high degree of stenosis; however, a different projection may reveal that the plaque’s encroachment into the lumen is far less severe, resulting in a lower apparent degree of stenosis (Figure 3). It is important to stress that these varying appearances do not reflect actual changes in the luminal surface area, which remains constant [29,42,43,44].

Projectional artifacts were common with the only one or two angiographic views of standard angiography, made multidirectional by adding oblique projections or three-dimensional acquisitions at the expense of additional radiation exposure. With CTA, these limitations are mostly solved with single-volume image acquisition, cross-sectional images, infinite projections, and 3D image volumes from computer post-processing.

The carotid bulb adds another layer of complexity to this assessment. This anatomical structure is an enlargement of the proximal part of the internal carotid artery and is a common site for atherosclerotic plaque formation. The bulb’s inherent widening can create challenges in accurately gauging stenosis severity, due to the difficulty of identifying a truly “normal” distal ICA segment where the walls are parallel for comparison, according to NASCET criteria, or the estimated original carotid bulb size, as per ECST [45,46,47].

### 4.2. Near-Occlusion

Carotid near-occlusion represents a progressed degree of severe carotid artery stenosis, characterized by partial or complete collapse of the distal ICA lumen caused by the stenosis [48,49,50]. The flow reduction caused by severe stenoses results in a smaller distal ICA (the collapse). It is fundamental to differentiate this condition from carotid “pseudo-occlusion”, which is typically caused by terminal intracranial ICA occlusion due to thromboembolism [50,51]. “String sign” [49] describes a very small distal ICA but is inappropriate as a synonym for near-occlusion because it also includes dissections but excludes near-occlusions with moderate distal ICA collapse (without full collapse).

The stroke risk associated with near-occlusion is lower compared to symptomatic severe conventional stenosis, i.e., those without near-occlusion [48,49,50], and the benefit of surgery is slight. The benefits of revascularization for symptomatic severe stenosis, as found in NASCET and ECST, are applicable only to severe stenoses without near-occlusion. Almost all (94%) near-occlusions in these trials were the moderate variant (without full collapse), so applying outcome results to those with full collapse is inappropriate [48,49,50].

Current guidelines recommend best medical therapy for carotid near-occlusion, though recent reviews question the superiority of medical therapy alone over carotid artery stenting or endarterectomy [47,52]. Distinguishing near-occlusion with or without full collapse has prognostic significance; those with full collapse have a higher risk of recurrent ipsilateral ischemic stroke or retinal artery occlusion within 28 days, while those with partial collapse have a lower recurrence risk [53]. Few near-occlusion cases with full collapse were randomized to NASCET/ECST due to a priori assumptions of worse surgical risks.

CTA has been the first-line modality for diagnosing carotid near-occlusion in recent years. Key features include a small extracranial ICA caliber compared to the contralateral ICA and external carotid artery and focal severe stenosis with minimal to no luminal contrast opacification [51,54].

**Figure 4:**
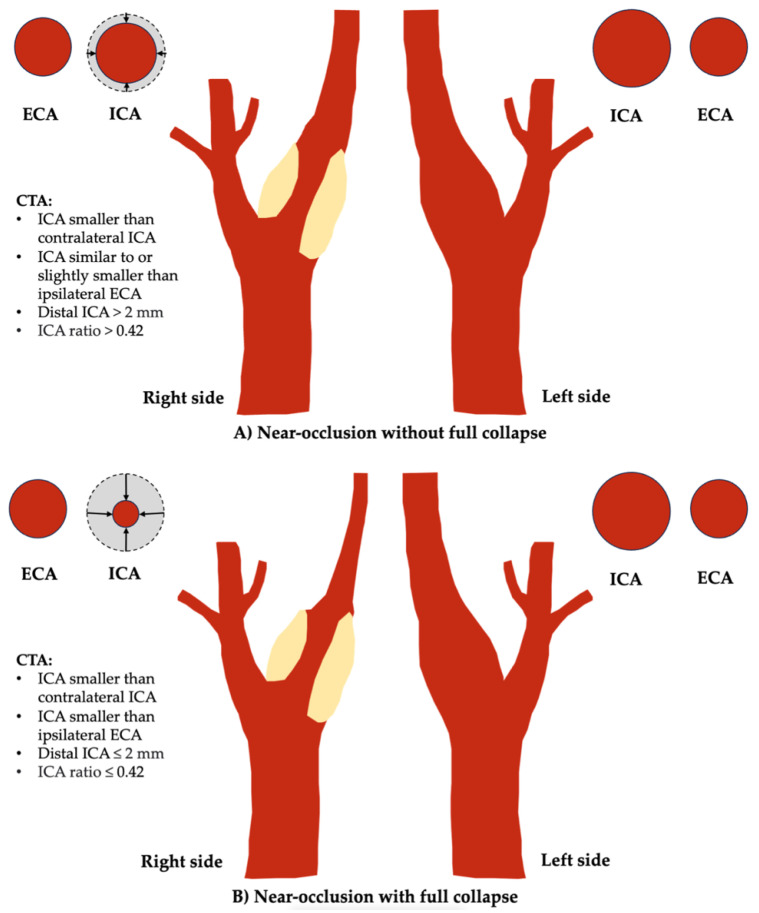
Schematic representation of near-occlusion with the recently proposed CTA identification criteria. (**A**) without full collapse on the left image; (**B**) with full collapse on the left image.

The diagnosis is set by systematic feature interpretation, assessing whether the distal ICA is small and whether proximal severe stenosis is the most reasonable cause [49,51]. While measurement criteria have been proposed, they have not been successfully validated [55,56]. As a result, near-occlusion remains an interpretative diagnosis arrived at by a skilled observer seeking the parameters. Full collapse traditionally appears as a threadlike residual lumen, but new, prognostic-driven criteria have been proposed for defining full collapse: distal ICA diameter ≤2.0 mm or an ipsilateral to contralateral distal ICA diameter ratio ≤0.42 (Figure 4) [53].

A main differential is anatomic variance, where the distal ICA is small due to Circle of Willis asymmetry [57]. Rarely, a hypo-developed ICA can occur and can be distinguished from complete near occlusion collapse by an associated small bony carotid canal [49].

As for US, it should be noted that, despite many studies on the topic [30,33,58,59,60], the approach to diagnose near-occlusion with ultrasound remains unclear. As a result, a large majority of near-occlusions are misdiagnosed with carotid ultrasound, as most near-occlusions have flow velocity in the stenosis, so near-occlusions with partial collapse are overlooked, and many near-occlusions with full collapse are not identified by a small distal ICA in B-mode, or are mistaken for occlusions [61,62]. A sonographic parameter warranting further investigation is distal PSV which, in high-PSV stenosis, has been found to differentiate near-occlusions from conventional ≥50% stenoses when found to be lower than 50 cm/s (63% sensitive and 93% specific) [63].

A recent innovation is the use of phase-contrast MRI, which distinguishes conventional stenosis and near-occlusion well [51]. Given that near-occlusions are difficult to diagnose, it is not surprising that many think that the condition is rare, but it actually represents nearly a third of cases with ≥50% stenoses [64].

Indeed, many seem to apply the percent stenosis calculation directly when grading with NASCET, skipping the first step of first excluding near-occlusions [65]. Compared to the experience from the assessment of the NASCET and ECST trials [62], near-occlusion has been more prevalent in consecutive series [62]; some of this is likely attributable to selection bias in trials, but a shift in recognizing more subtle near-occlusions in recent years cannot be excluded [66].

For radiology reporting, near-occlusion should be distinguished from severe stenoses without near-occlusion. Percent stenosis is fallacious in near-occlusion cases; reports should specify the grade of stenosis as “near-occlusion,” indicating whether there is full or partial collapse [25,47,48,49,50]. Here, it is important to realize that while it is intuitive to think of stenoses as on a spectrum from 0–100%, within the NASCET itself, when near-occlusion was prospectively identified, no measurements were taken or percentage stenosis calculated as that would have been wrong. For data analysis at the time, a symbolic entry permitted analysis of all NASCET cases.

A summary of the different proposed near-occlusion criteria in Table 3.

### 4.3. The NASCET Pitfalls

The NASCET method for measuring carotid stenosis, although described in a standardized way, is fraught with complexities and susceptibility to errors, particularly in routine clinical practice [9,10].

The first pitfall consists of correctly recognizing near-occlusion first to avoid fallacious measuring of partly or fully narrowed ICA beyond a stenosis; the second is correctly identifying distal ICA well beyond the tapering bulb, to a level where the walls are parallel.

Despite these well-known situations, in practice, many practitioners either mistakenly choose points that are too proximal, where the artery is still tapering, and apply the formula incorrectly, or are tempted to use the so-called “eyeball method”, with the result of over-stating the stenosis severity [44,45]. The extra work to actually do the measurement as defined by NASCET [67], avoiding the pitfalls, will usually yield a stenosis less severe in % stenosis than if done by eye or without paying attention to the pitfalls, therefore less often meaning that a patient’s stenosis qualifies for surgical intervention according to the NASCET criteria. In effect, the NASCET outcome results, with strong indications for surgical intervention, became conscious or subconscious reasons to declare higher stenoses percentages than NASCET might have done [68].

Another concerning problem is interobserver variability. Different practitioners, including experienced vascular surgeons and radiologists, may have varying interpretations when assessing arteriograms: studies have indicated notable discrepancies in these interpretations, yet these measurements form the basis of critical clinical decisions [16,45]. Although some research has reported commendable interobserver and intraobserver agreement using the NASCET criteria [69,70], such results are not consistently replicable across different clinical environments with diverse levels of expertise and experience.

The consequences of these measurement inaccuracies are potentially far-reaching and serious. Inaccurate calculations can lead to overestimation of the severity of carotid stenosis [71,72]; as a result, patients may be advised to undergo unnecessary surgical interventions such as carotid endarterectomy (CEA) or carotid artery stenting (CAS). These procedures are not risk-free and carry potential complications, including stroke, cranial nerve injuries, and even death. The overuse of surgical interventions due to miscalculated carotid stenosis exposes patients to unnecessary health risks and it also contributes to inflated healthcare costs. Additionally, the psychological impact on patients who are incorrectly informed about the severity of their condition cannot be understated. The stress and anxiety associated with undergoing an unnecessary surgical procedure, along with the potential for postoperative complications, can significantly affect a patient’s quality of life [73,74].

Moreover, this issue extends beyond individual patient care. Inaccurate application of the NASCET criteria can skew the data and findings of clinical studies, leading to flawed conclusions and recommendations. This, in turn, affects broader medical understanding and guidelines regarding the management of carotid stenosis.

### 4.4. Diameter and Area

The linear diameter of the narrowest visible stenosis was always the main part of the ratio and the parameter measured by the direct mm approach [18]. Cross-sectional area, which can be measured by pixel-counting digital tools, has been investigated as an alternate method to quantify carotid stenosis, considering that plaques can be irregularly shaped and noncircular. Bartlett compared direct mm with area [20] and found no significant advantage in pixel-counting for area.

**Figure 5:**
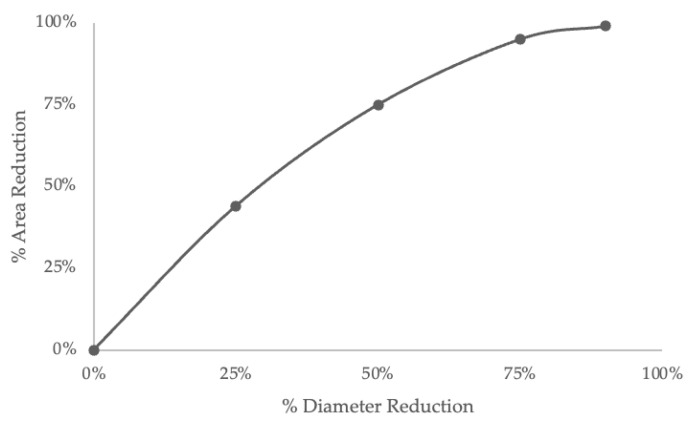
Graph illustrating the relationship between area reduction and diameter reduction in a completely concentric stenosis.

The distinction between the measurement of stenotic area and the degree of stenosis based on the ratio of diameters transcends a mere terminological discrepancy: while the degree of stenosis calculated using diameters considers the luminal narrowing in a linear dimension, the measurement of stenotic area accounts for a two-dimensional reduction of the vascular lumen and changes in the area are quadratic in relation to changes in the diameter (Figure 5) [29].

Different studies have focused on this topic, with conflicting results. On one side, studies claim no difference between diameter-based and area-based measurements [20,23]; on the other side, working groups find higher accuracy for carotid stenosis with CTA area-based measurements [75,76]. The question of whether area stenosis will be better than diameter stenosis, or similar, in the prediction of ischemic risk for thromboembolism needs further study. The factors that predict small thrombus formation include eddy currents from the distortion of laminar blood flow through stenoses [77,78,79]. Whether the narrowest part induces thrombus-inducing stresses similar to or more than an asymmetrical plaque is uncertain. The linear diameter may not ultimately be different in importance, as suggested by Bartlett [20], but may or may not be valid clinically, and needs study.

There is a dilemma about how to proceed at this time. The trials first completed for severe stenosis in 1991 analyzed, as consistently as possible, the angiographic films submitted to the study core centers. The outcome results analyzed, especially the high stroke rates for the most severe stenoses using NASCET and the high efficacy of surgery, were based on the % stenosis NASCET method. We now are aware that the pitfalls of utilizing the NASCET method have meant it has been inconsistently used to create % stenosis in the 32 years since; near-occlusions are now recognized in higher numbers when sought after, affecting the identification of cases. Angiography is now mainly performed with stroke-risk-free CTA, exacting mm measurements of stenosis diameters are made, and ratios can be calculated, but still with the original pitfalls. Bartlett’s converted mm stenosis to % NASCET ratios published in 2006 suggest that denominators and ratios are no longer needed.

Conventionally, it might be said that until validation studies of Bartlett’s work, seemingly ignored since 2006, are carried out, the degree of stenosis calculated as a ratio of diameters, as utilized in NASCET and ECST criteria, might be the preferred and most clinically substantiated method for guiding therapeutic decisions. However, we also know that the film analyses done in 1991 were crude based on the high-level CTA detail today. It seems prudent then that CTA, performed as stroke risk-free angiography, should be done, and mm measures be taken and recorded, as a working hypothesis. The linear measurement of stenosis should be recorded, as well as any denominator measurement, for % stenosis calculation to be continued while waiting for validation of the Bartlett data. It is still paramount for clinicians to be cognizant of these distinctions and employ evidence-based approaches when evaluating and managing patients with carotid artery stenosis [24].

## 5. Discussion and Potential Future Directions

### 5.1. The Changing Landscape: The Carotid Vulnerable Plaque

Historically, the extent of arterial narrowing was regarded as the primary indicator for assessing stroke risk severity. This perspective was mainly informed by studies from the 1980s and 1990s, which highlighted the effectiveness of carotid endarterectomy (CEA) in patients with substantial stenosis (ranging from 70% to 99%). Key trials contributing to this understanding included the NASCET [9,10], the ECST [11,12], and the Asymptomatic Carotid Atherosclerosis Study (ACAS) [80], alongside the Asymptomatic Carotid Surgery Trial (ACST-1) [81].

ACAS and ACST-1 compared CEA plus best medical therapy (BMT) alone in patients with asymptomatic carotid artery stenosis (aCAS) >60% according to NASCET assessment: ACAS found a 5-year absolute risk of ipsilateral stroke, perioperative stroke, or death of 5.1% vs. 11% in the CEA vs. BMT arm; ACST-1 found a 5-year risk of ipsilateral stroke, perioperative stroke, or death of 6.4% vs. 11.8% in the CEA vs. BMT arm, prompting a discussion of whether CEA is beneficial in asymptomatic patients with a high degree of carotid stenosis and of the indication for CEA in asymptomatic patients with >70% stenosis in the 2011 guidelines of the AHA and other 13 societies [82].

However, recent viewpoints challenge the traditional risk–benefit assessment of CEA, particularly for patients with symptomatic plaques devoid of high-risk biomarkers like intraplaque hemorrhage (IPH). This reconsideration stems from advancements in medical therapies [83,84,85,86,87].

The discussion becomes even more complex when considering asymptomatic patients with over 50% stenosis. Analysis by Abbott and colleagues showed significant declines in reported rates of ipsilateral stroke/TIA and any-territory stroke/TIA from 1985 to present, underscoring the efficacy and cost-effectiveness of medical management over combined medical/surgical intervention in asymptomatic stenosis [88], with ongoing trials such as the Carotid Revascularization and Medical Management for Asymptomatic Carotid Stenosis study (CREST-2) [89] and ECST-2 [90] re-examining this aspect.

Despite these insights, consensus on exclusively using medical therapy for asymptomatic carotid stenosis is yet to be reached. Several studies have pointed out the role of non-stenosing carotid artery plaques in a considerable number of cryptogenic strokes. Research by Kopczak et al. on 234 patients revealed a notably higher prevalence of complex carotid artery plaques (CAP) on the side of the brain corresponding to the infarct in patients with cryptogenic stroke (CS) compared to the opposite side [91]. CAP prevalence was significantly greater in CS compared to cardioembolic/small vessel stroke (CES/SVS), with larger lipid-rich necrotic cores in ipsilateral CAP in CS compared to CES/SVS.

Further, their subsequent study highlighted that in patients with cryptogenic stroke and a complex, non-stenotic carotid plaque on the side corresponding to the ischemic territory, there was a substantially higher risk for recurrent stroke or TIA. The 3-year incidence rate of TIA/stroke was markedly higher in these patients compared to those without such plaques at the outset [92].

In the ACSRS cohort of 1121 patients with asymptomatic carotid stenosis, groups at high annual stroke risk (>4%) were identifiable using ultrasonographic plaque texture features. However, these patients were not on what is currently considered optimal medical therapy [93].

Given this diverse landscape, an increasing body of literature suggests that recognizing unstable plaque characteristics might substantially improve the evaluation of carotid atherosclerosis in both symptomatic and asymptomatic individuals. This approach potentially offers greater predictive accuracy than stenosis measurement alone and aids clinicians in precisely tailoring medical or surgical interventions [94,95]. Identifying high-risk components in patients already receiving optimal medical care might indicate the necessity of revascularization in asymptomatic individuals or support a less invasive approach in symptomatic patients unsuitable for surgery.

To effectively apply current knowledge on plaque vulnerability in clinical practice, a standardized reporting system is crucial. This standardization will minimize variability in terminology and classification across institutions, ensuring clear and systematic information exchange between imaging specialists and referring doctors.

In the 1990s, the American Heart Association (AHA) introduced a classification for atherosclerotic lesions, primarily focused on coronary artery plaques, to provide a histological reference for images acquired via invasive and non-invasive methods [96]. This classification was later updated in 2000. The AHA system assigns Roman numerals (I to VIII) to lesion types, with an order reflecting the expected progression sequence. While the initial types (I-III) are typically small and clinically silent, types IV-VI can obstruct the lumen or trigger clinical events. Types VII and VIII are characterized by fibro-calcific changes. Notably, the clinical manifestation of disease can vary, meaning that a type-IV lesion (atheroma with confluent extracellular lipid core) can progress to type-VI changes (complicated plaque) without transitioning through a type-V stage (fibroatheroma) [97].

Cai and colleagues demonstrated MRI’s capacity to characterize carotid plaque components following the AHA classification, with significant agreement between MRI findings and AHA categories, leading to a proposed modified AHA classification for MRI [98]. While the AHA classification has been a reference for decades, its practical clinical application has been limited due to its histology-centric approach and focus on lesion natural history rather than clinical urgency. For instance, a type-VIII lesion in this system indicates a less severe/stable lesion than a type-VI lesion, considered a high-risk vulnerable plaque.

Recently, in October 2023, to further expand upon this point, the Carotid Plaque RADS was introduced [99], offering a reliable multi-imaging scoring reporting system (1) to provide the risk of cerebrovascular events based on carotid plaque morphology and (2) to facilitate data mining and research with standardized terminology.

**Figure 6:**
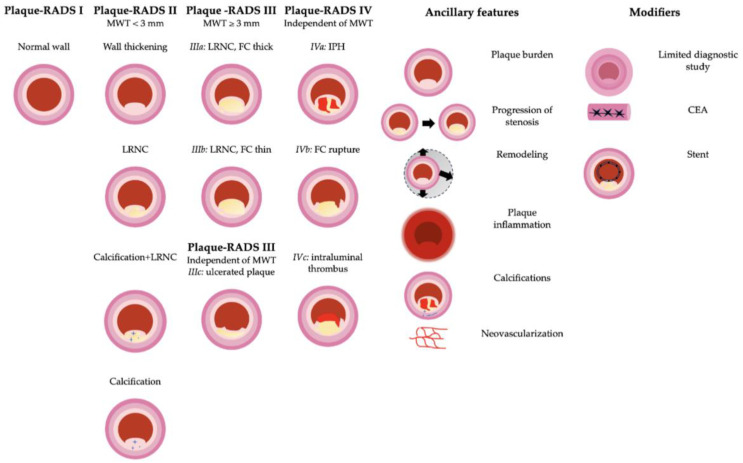
Schematic representation of the different Carotid Plaque RADS categories from 1 to 4, ancillary features, and modifiers. Presentation concept based on Saba et al. [96].

Specifically, the Carotid Plaque RADS (Figure 6) considers:

- Imaging features: maximum wall thickness (MWT), lipid-rich necrotic core (LRNC), intraplaque hemorrhage (IPH), fibrous cap (FC) rupture, and intraluminal thrombus;
- Ancillary features: plaque inflammation and neovascularization, positive carotid remodeling, plaque burden, progression of stenosis, and carotid plaque calcifications;
- Modifiers: limited diagnostic study (L), stents, and previous CEA.

Normal vessels with normal vessel walls and absence of plaque define plaque-RADS 1.

MWT < 3 mm, without imaging features of complicated plaques, defines plaque-RADS 2, which is a relatively stable plaque and therefore a low-risk plaque, mainly consisting of fibrous tissue, small lipid pools, and a small lipid-rich necrotic core (LRNC), but also a potential precursor of complicated plaques, should its components modify over time [100].

In plaque-RADS 3, there is an MWT ≥ 3 mm, a moderate-to-large LRNC, calcifications, healed ulcerations, and calcifications. The subcategories 3a, 3b, and 3c are defined by the presence of thick FC, thin FC, and plaque ulceration, respectively. LRNC has been associated with an increased risk of future ipsilateral cerebrovascular events in a meta-analysis by Gupta et al. [101].

IPH, characteristic of category 4 and first reported by Moody et al. in 1999 [102], is the most common feature of complicated plaques. It has been object of extensive research [103,104], being present in 89% of all complicated plaques ipsilateral to acute ischemic stroke as demonstrated by Kopczak and colleagues [91] and associated with recurrent stroke, as indicated by the recent prospective Plaque at Risk (PARISK) study [105].

Fibrous cap (FC) rupture, which is present in categories 4b and 4c, forms part of a dynamic process along with thrombus formation, healing, and remodeling of the plaque [106], and is also linked to cerebrovascular events [101].

The intraluminal thrombus, defining category 4c, is a recognized predictor of stroke of carotid origin and is found in up to 92% of patients with neurologic symptoms [100,107,108,109].

The ancillary features represent validated features of plaque instability that do not determine the main Plaque-RADS score but can represent a complementary tool if present. These include inflammatory changes in the perivascular fat [110,111], carotid artery remodeling [112,113], plaque burden [114,115], progression of stenosis [116], and carotid plaque calcification, in particular the positive rim sign [117,118,119].

The modifiers, as in CAD-RADS [120], can be complemented to indicate the situation where the study is not fully diagnostic, or where the presence of metal-induced artifacts may influence the correct assessment of the carotid plaque morphology.

US can evaluate intima-media thickness (IMT), wall thickness, plaque volume [121,122,123,124,125], and thick fibrous cap (FC) [126], the latter presenting as an hyperechoic structure and being the hallmark of the score 3a.

The echogenicity of LRNC cannot be distinguished from IPH on US, and so-called juxta-luminal black areas (JBA), consisting of LRNC with a thin FC and defining score 3b, can also be due to plaque rupture (score 4b) or intraluminal thrombus (score 4c) [127,128].

As a result, US is sufficiently diagnostic for the plaque-RADS categories 1, 2, and 3a; however, if a hypoechoic plaque without a visible hyperechoic FC or a JBA is seen on US, additional imaging is required. In this context, MRI is preferred since it is widely accepted that, given the overlapping HU, CT cannot adequately differentiate between LRNC and IPH; nor can it determine FC thickness and/or integrity.

On MRI, IPH is identified as a focus of hyperintensity in the context of the plaque on heavily T1- weighted images, with an inversion pre-pulse to suppress the signal of blood show (MPRAGE) [129], while FC thickness is detected on time-of-flight (TOF) images as a band of low signal, if present [130].

Each imaging modality, US, CT, or MRI, has its limitations.

US is operator-dependent and is limited by the presence of shadowing due to calcifications and patient-related anatomical considerations [27,40,131].

CT, besides the well-known exposure to ionizing radiation and adverse reactions to the iodinated contrast medium, cannot distinguish completely between IPH, LRNC, and fibrous tissue due to overlapping Hounsfield Units (HU) [132].

Lastly, MRI is limited due to its extended acquisition periods, low availability, and requirement for specialized coils [133].

### 5.2. Photon-Counting CT (PCCT)

As previously stated, CT plaque characterization is limited due to the overlapping HU values of some of its components. In this regard, photon-counting CT has the potential for overcoming these limitations, owing to its capacity to generate quantitative maps depicting the spatial distribution of atomic elements within a plaque [134].

In a recently published study by Dahal et al. [135], FC thickness, FC area, and LRNC were identified and quantified through PCCT technology, confirming results already observed in cardiac imaging [136,137].

### 5.3. AI Impact

Artificial intelligence (AI) can be defined as the general ability of computers to emulate human cognitive processes and perform tasks [138].

Machine learning (ML) is a subfield of AI, solving problems by extracting patterns from raw data without explicit programming. In deep learning, a subset of ML, artificial neural networks mimic the learning process of the human brain [138,139,140].

The applications of AI in carotid atherosclerotic disease in the different diagnostic modalities range from automatic carotid lumen segmentation to automatic carotid plaque segmentation and automatic detection of vulnerable plaque [141,142,143,144,145,146,147,148], potentially helping in implementing the novel Plaque-RADS score in clinical practice [99].

It is important to note that while AI holds great promise in aiding the health-care professional to optimize patient care, its application is constrained by data heterogeneity, the limited explainability of numerous AI algorithms, a lack of external validation in the current literature, and, last but not least, ethical and regulatory considerations.

To facilitate the incorporation of AI-based models into routinary clinical practice it is essential to develop prospective and multicenter studies with clear and open-access designs.

A growing interest is emerging in computational fluid dynamics (CFD) to simulate blood flow inside the carotid arteries [149,150]. Indeed, the hemodynamic environment affects development as much as progression and plaque complications [77,78,79].

This has already been used in coronary disease [151] and intracranial atherosclerotic disease, with promising results [152,153].

## 6. Conclusions

NASCET and ECST are historical studies on carotid disease. Their results provided clear guidelines for clinical decision-making. They also highlighted the need for standardization of imaging techniques and of the quantitation of carotid stenosis in clinical and research settings.

With advances in CTA, new quantification methods, such as the direct mm approach by Bartlett, have emerged, enabling clinical and research contexts for measuring stenosis directly, rather than calculating ratios.

It is crucial to focus research on clinical trials linking the newer techniques to the old ones and to specific therapeutic choices in order to eventually update the guidelines.

Moreover, it remains crucial to identify and report those features suggestive of plaque vulnerability, along with stenosis quantification, to better stratify patients and tailor their treatment accordingly.

In this regard, AI and new CT technologies will play a role in helping to overcome the challenges radiologists currently face in their clinical routines.

## Figures and Tables

**Table 1:** The relationship between ECST and NASCET % stenosis. The conversion scale is based on the following equations: NASCET = (ECST − 40) %/0.6 and ECST = 40 + (0.6 × NASCET %).

ICA Stenosis (%NASCET)	ICA Stenosis (%ECST)
30	60
50	70
60	75
70	80
80	90

**Table 2:** Correlation between the NASCET method and US.

ICA Stenosis (NASCET)	ICA PSV (cm/s)	ICA EDV (cm/s)	PSV Ratio (ICA/ECA)
Normal	<125	<40	<2.0
<50%	<125	<40	<2.0
50–60%	125–130	40–100	2.0–4.0
>70%	>230	>100	>4.0
Near-occlusion	Variable	Variable	Variable
Total occlusion	Undetectable	Undetectable	Undetectable

Adapted from Grant et al. [30].

**Table 3:** Examples of proposed near-occlusion criteria according to different diagnostic techniques.

DSA [48]	Delayed time of contrast arrival Evidence of collaterals ICA-to-ICA comparison of diameter reduction ICA-to-ECA comparison of diameter reduction
US [63]	Distal PSV < 50 cm/s in high-PSV stenoses
CTA [53]	Distal ICA < 2 mm ICA ratio < 0.42
PC-MRA [51]	ICA-CBF * < 0.225

* ICA-CBF: Ratio between ICA and CBF, where CBF = ICA-ipsilateral + ICA-contralateral + BA. ICA: internal carotid artery; CBF: cerebral blood flow; BA: basilar artery.

## Data Availability

No new data were created or analyzed in this study. Data sharing does not apply to this article.
